# Targeting Medication Non-Adherence Behavior in Selected Autoimmune Diseases: A Systematic Approach to Digital Health Program Development

**DOI:** 10.1371/journal.pone.0129364

**Published:** 2015-06-24

**Authors:** Trevor van Mierlo, Rachel Fournier, Michael Ingham

**Affiliations:** 1 Evolution Health Systems Inc., 1266 Queen Street West, Suite 8, Toronto, Ontario, M6K 1L3, Canada; 2 Research Associate, Henley Business School, University of Reading, Greenlands, Henley-on-Thames, Oxfordshire, RG9 3AU, United Kingdom; 3 Janssen Scientific Affairs LLC, 850 Ridgeview Dr., Horsham, Pennsylvania, 19044, United States of America; University of Birmingham, UNITED KINGDOM

## Abstract

**Background:**

29 autoimmune diseases, including Rheumatoid Arthritis, gout, Crohn’s Disease, and Systematic Lupus Erythematosus affect 7.6-9.4% of the population. While effective therapy is available, many patients do not follow treatment or use medications as directed. Digital health and Web 2.0 interventions have demonstrated much promise in increasing medication and treatment adherence, but to date many Internet tools have proven disappointing. In fact, most digital interventions continue to suffer from high attrition in patient populations, are burdensome for healthcare professionals, and have relatively short life spans.

**Objective:**

Digital health tools have traditionally centered on the transformation of existing interventions (such as diaries, trackers, stage-based or cognitive behavioral therapy programs, coupons, or symptom checklists) to electronic format. Advanced digital interventions have also incorporated attributes of Web 2.0 such as social networking, text messaging, and the use of video. Despite these efforts, there has not been little measurable impact in non-adherence for illnesses that require medical interventions, and research must look to other strategies or development methodologies. As a first step in investigating the feasibility of developing such a tool, the objective of the current study is to systematically rate factors of non-adherence that have been reported in past research studies.

**Methods:**

Grounded Theory, recognized as a rigorous method that facilitates the emergence of new themes through systematic analysis, data collection and coding, was used to analyze quantitative, qualitative and mixed method studies addressing the following autoimmune diseases: Rheumatoid Arthritis, gout, Crohn’s Disease, Systematic Lupus Erythematosus, and inflammatory bowel disease. Studies were only included if they contained primary data addressing the relationship with non-adherence.

**Results:**

Out of the 27 studies, four non-modifiable and 11 modifiable risk factors were discovered. Over one third of articles identified the following risk factors as common contributors to medication non-adherence (percent of studies reporting): patients not understanding treatment (44%), side effects (41%), age (37%), dose regimen (33%), and perceived medication ineffectiveness (33%). An unanticipated finding that emerged was the need for risk stratification tools (81%) with patient-centric approaches (67%).

**Conclusions:**

This study systematically identifies and categorizes medication non-adherence risk factors in select autoimmune diseases. Findings indicate that patients understanding of their disease and the role of medication are paramount. An unexpected finding was that the majority of research articles called for the creation of tailored, patient-centric interventions that dispel personal misconceptions about disease, pharmacotherapy, and how the body responds to treatment. To our knowledge, these interventions do not yet exist in digital format. Rather than adopting a systems level approach, digital health programs should focus on cohorts with heterogeneous needs, and develop tailored interventions based on individual non-adherence patterns.

## Background

### Digital Health Interventions

In the mid-1980’s research began to examine how digital technology could be facilitate improved patient health [1,2]. Since then, evidence has indicated that many patients would prefer to communicate with their physician via email [3], that patient-led Internet support systems can safely help with complex behavioral issues [4], and that physicians, payers and hospital systems could benefit by leveraging the Internet to improve communication amongst stakeholders [5]. Several Cochrane reviews have indicated that digital health programs can be a benefit to patients [6–10], however to our knowledge, the literature has not identified effective digital programs for autoimmune disorders.

Digital health tools have traditionally centered on the transformation of existing paper-based interventions (such as diaries, trackers, stage-based or cognitive behavioral therapy programs, web-based coupon downloads, or symptom checklists) to electronic format. Advanced digital interventions have also incorporated attributes of Web 2.0 such as social networking, text messaging, and the use of video. However, Internet interventions typically have high attrition and have so far failed to produce population effects [11–13].

To further complicate matters, failures to deliver effective systems, tools and interventions have been reported in countries traditionally associated with high Internet adoption rates. Countries such as Australia [14,15], Canada [16,17], the United Kingdom [18,19] and the United States [20–22] have all reported massive eHealth failures, and the potential of digital health remains unrecognized.

### The Adherence Problem

A well-known, systemic problem in healthcare is medication non-adherence. The World Health Organization estimates that the average non-adherence rate in developed countries is 50% among patients with chronic conditions [23]. Other studies estimate that non-adherence in North American accounts for $300 billion dollars in avoidable costs, annually [24–26].

Non-adherence is a complex problem. At the patient level, medication non-adherence is generally defined as *intentional* or *non-intentional* [27]. Strongly associated with personal beliefs and perceptions, patients actively choose to ignore treatment in intentional non-adherence, whereas unintentional non-adherence involves a passive process [28,29]. Risk factors in treatment are well reported in the literature [30–32] and are generally labeled as *modifiable* (behavior-based) or *non-modifiable* (eg. age, gender).

Identifying a patient’s unique non-adherence patterns is time consuming, and existing paper-based processes that require scoring, are complex and often expensive to administer.

### Digital Health Interventions and Non-Adherence

One of the most attractive aspects of digital health is the ability to use algorithms to tailor interventions to individual preferences [33–35]. However, and as mentioned previously, most interventions are based on traditional approaches designed for large homogenous populations.

Rather than adopting a systems-level approach, digital health might focus on cohorts with heterogeneous needs, and develop tailored interventions based to individual non-adherence patterns.

To our knowledge, publicly-available digital health tools designed to target specific non-adherence behaviors in autoimmune diseases do not exist. As a first step in investigating the feasibility of developing such a tool, the objective of the current study is to systematically rate factors of non-adherence that have been reported in past research studies.

Although several risk factors are well known (cost, forgetfulness, co-morbidities), they have yet to be grouped, ranked or systematically categorized. To uncover common risk factors observed by researchers, we systematically reviewed qualitative, quantitative and mixed-methods literature that reported factors noted in their respective cohorts.

Recognized as a rigorous method to facilitate the emergence of common themes in previously published literature, we used Grounded Theory (GT) as process to uncover these factors [36]. Before describing the specific GT methodology used in our research, an outline of the magnitude of non-adherence among autoimmunology stakeholders will be first presented.

### The Adherence Problem

Approximately 8–9% of the population is affected by 29 autoimmune diseases, including Rheumatoid Arthritis (RA), gout, Crohn’s Disease (CD), and Systemic Lupus Erythematosus (SLE) [37]. As is typical with chronic conditions, many patients do not adhere to long-term therapies. In autoimmunology, reported estimates of non-adherence in research studies range from 7–84% [38–40]. This wide range can be attributed to difficulties in both recording individual rates of non-adherence, and a lack of measurement standards.

Traditional research methods used to measure medication adherence include patient self-report, Electronic Monitoring (EM), and direct observation. Although EM is most accurate, patient self-report and EM may be unreliable as they are potentially subject to the Hawthorne effect [41] (i.e., adherence behavior changes as a result of being monitored, and some researchers choose to not inform patients of monitoring to avoid the observation effect).

In retrospective database studies, adherence is most commonly reported in terms of Medication Possession Ratios (MPR) and/or Proportion of Days Covered (PDC). Deriving MPR and PDC requires access to patient-level treatment utilization data, which is typically retrospective. MPR and PDC are not necessarily accurate, as it cannot be guaranteed that patients are following treatment simply because they are fulfilling prescriptions or collecting medication at expected intervals [42].

The only current means to ensure adherence is by direct observation without patient knowledge [43,44]. Unfortunately, this method is difficult to replicate and perform on a large scare, so true non-adherence rates may be impossible to estimate.

### Costs Related to Non-Adherence

As in other chronic conditions, non-adherence rates in autoimmunology lead to increased costs throughout healthcare systems. Costs resulting from the progression of disease and the subsequent need for more aggressive treatment also result in significant economic burden [45], and when immunological diseases are comorbid with other conditions, non-adherence to proven treatment increased health risks and costs for both disorders [46].

Likewise, adherent patients contribute to positive health benefits and economic outcomes. For example, a cohort study of 834 SLE patients found that decreased adherence led to increased visits to rheumatologists, primary care physicians, other care provides, emergency departments, and hospitalizations [47]. While adherence resulted in shorter hospital length of stay and lower inpatient costs in CD patients in one study [48], another discovered that when compared to adherent ulcerative colitis patients, those who were non-adherent incurred twice the inpatient costs and significantly greater overall health care costs [49].

## Methods

### Rigorously reviewing literature with Grounded Theory

Founded by Glaser and Strauss in 1967, GT is an objectivist methodology used to develop theoretical interpretations without defining phenomenon *a priori* [50]. In conducting GT, researchers first familiarize themselves with prior research to determine a general research question, followed by a mechanistic review and classification of data [51]. As it is a research method involving discovery through systematic analysis, data collection and coding [52], past studies have recognized GT as a method to study social psychological themes across diverse chronic illnesses [53].

**Fig 1:**
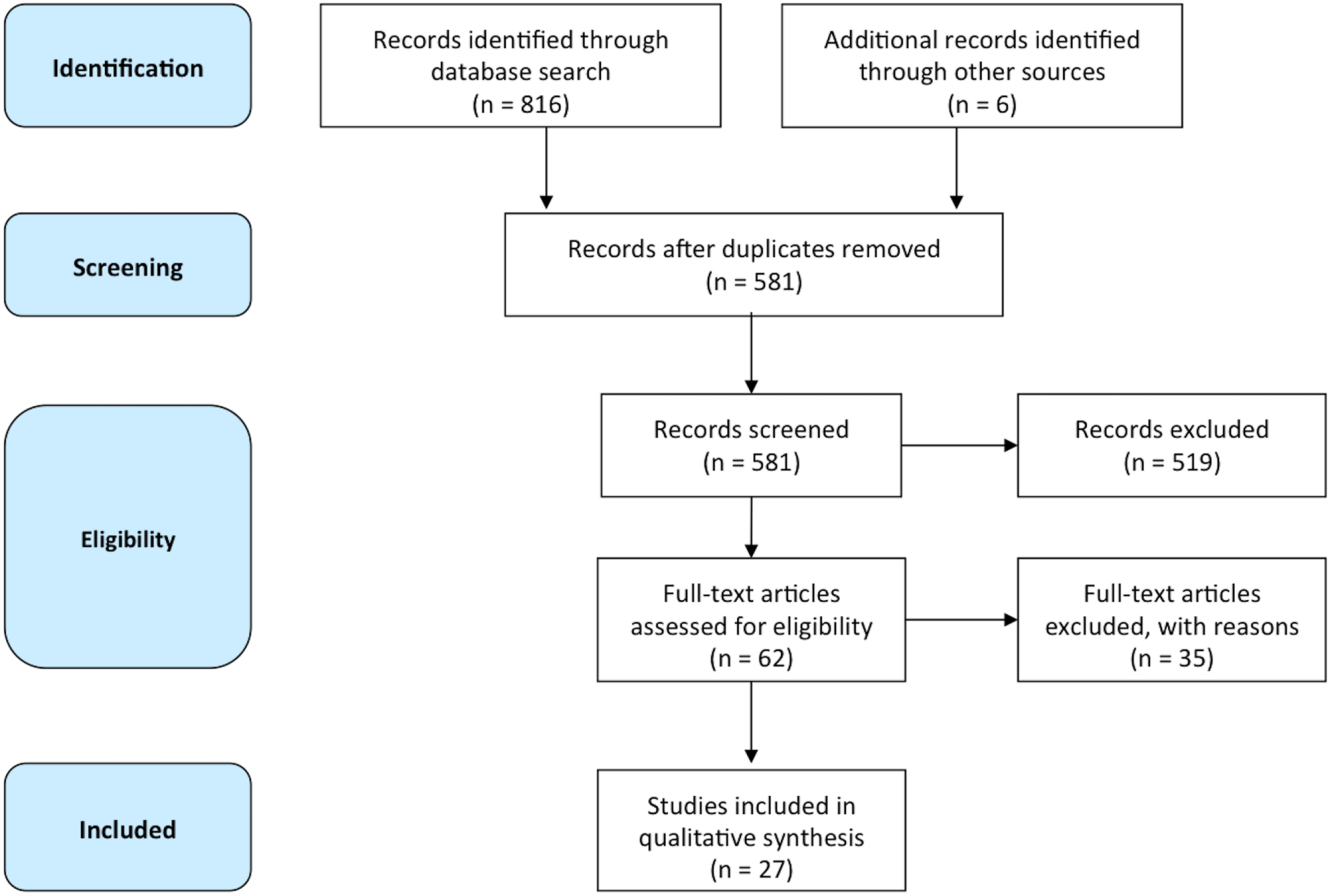
PRISMA flow diagram.

To uncover themes in previously published research, this study utilized the five-stage process outlined by Wolfswinkel et al. to conduct a rigorous literature review (*Define*, *Search*, *Select*, *Analyze*, *Present*) [36]. When followed correctly, this process assures an in-depth analysis of empirical facts, interrelationships and dependencies beyond a particular area, and the emergence of themes. In addition, this review followed the Preferred Reporting Items for Systematic Reviews and Meta-Analyses (PRISMA) checklist for systematic reviews [54]. The PRISMA flow diagram is provided in Fig 1. The PRISMA checklist is provided in S1 PRISMA Checklist.

#### Database and search methodology (*Define*, *Search*)

In the *Define* stage, we conducted a general literature review of various issues related to medication non-adherence over a number of chronic disease conditions. Initial findings primarily centered on recurring problems related to lack of defined terminologies (adherence, compliance, persistence, concordance) [55,56], delivery mechanisms (oral, subcutaneous, infusion), dose measurement [42], the doctor-patient relationship [57], and the ineffective nature of existing interventions [58,59].

During the S*earch* stage, the databases PubMed and Google Scholar were searched in May 2013. PubMed was selected as it is a standardized database frequently referenced by physicians to seek information, and Google Scholar was utilized as it has recently been identified as an accurate reference for quick clinical searches [60].

As mentioned previously, there are 29 autoimmune diseases, and the literature on all of these disorders is vast. Likewise, the research on non-adherence and related terminology (e.g. compliance, concordance, discordance, persistence, shared decision-making, the therapeutic alliance), delivery mechanisms and does regimen is also voluminous. In order to gain insight on the research question and to simplify the search process, we limited MeSH keywords to a subset of autoimmune disorders.

The keywords *rheumatoid arthritis*, *rheumatology*, *gout*, *crohn’s disease*, *systematic lupus erythematosus*, *inflammatory bowel disease* and *medication adherence* were used in the search process. The initial database search returned 816 records. Following the removal of duplicates, 581 abstracts were included for further analysis.

#### Study selection (*Select)*

For the purpose of knowledge generation, meta-analyses, Cochrane reviews, case studies, reports, letters to the editor and opinion articles were reviewed in the initial screening process, but to synthesize results and remove the possibility of replication, were excluded in the coding process.

Fig 1 presents the PRISMA flow chart for the selection of included studies. Articles were included for eligibility coding if they met the following criteria:

1. Peer-reviewed studies reporting primary data only.
2. Quantitative (e.g. as measured by MPR or PDC), qualitative (e.g. through the use of surveys or interviews), and mixed-methods studies that measured adherence within autoimmunology indications.
3. Articles in English language only
4. Any jurisdiction or geographical location.

Study selection was conducted in two stages—initial screening (TvM) and subsequent validation by a second researcher (RF). The first researcher screened the 581 abstracts, confirming 519 did not meet study criteria, resulting in 62 potentially relevant studies. The *Select* process then required a full examination of the 62 articles. Following full examination, a further 34 articles were discarded, leaving 28 studies that met all inclusion criteria. Double-checking this process with the second researcher resulted in the further exclusion of one additional study, leaving 27 studies.

#### Coding of studies (*Analyze)*

As recommended by Wolfswinkel et al, open, axial and selective coding was applied systematically to all included studies. In open coding, researchers reviewed the 27 studies to develop and categorize meta-insights and concepts. Following this, all studies were re-reviewed and re-coded to investigate the consistency of meta-insights and themes (axial coding), resulting in the establishment of main themes and patterns. Finally, researchers performed selective coding where theoretical saturation of themes and patterns was established. As outlined in GT study protocol, a reliability check was conducted to confirm risk factor identification, coding procedure, scoring, and results [61]. Each study was coded with NVivo qualitative data analysis software, version 10.

Risk factors were only coded if they were explicitly noted in the Results, Discussion or the Conclusion section of each study. If a risk factor was identified in a study it received a score of one. Even if the risk factor was noted numerous times in each study, the maximum score a risk factor could receive in a single study was one (Table 1).

**Table 1:** Summary of Studies Reporting Factors of Non-Adherence (n = 27).

Citation	Research Method/Sample	Country of Origin	Disease	Non-Adherence Factor(s) Listed / Recommended Approach
Bermejo et al., 2010	Survey (N = 107)	Spain	Inflammatory bowel disease	Patient does not understand treatment, Forgetfulness or inconvenience, Dose regimen Lack of motivation or social support, Risk stratification tool or intervention recommended, Patient-centric approach recommended
Cannon et al., 2011	Retrospective database (N = 1412)	USA	Rheumatoid arthritis	Ethnicity, Gender (females less adherent), Disease severity, Risk stratification tool or intervention recommended
Dalbeth et al., 2012	Cross sectional assessment (N = 273)	New Zealand	Gout	Ethnicity, Gender (males less adherent), Patient does not understand treatment, Lack of motivation or social support, Disease severity, Risk stratification tool or intervention recommended, Patient-centric approach recommended
de Turrah et al., 2010	Longitudinal study (N = 941)	Denmark	Rheumatoid arthritis	Disease duration, Perceived medication ineffectiveness, Disease severity, Comorbid conditions, Risk stratification tool or intervention recommended
de Turrah et al., 2010	Retrospective database, questionnaire (N = 126)	Denmark	Rheumatoid arthritis	Disease duration, Patient does not understand treatment, Perceived medication ineffectiveness, Risk stratification tool or intervention recommended
Daleboudt et al., 2011	Questionnaire (N = 106)	New Zealand	Systemic lupus erythematosus	Age, Ethnicity, Patient does not understand treatment, Side effects Forgetfulness or inconvenience, Mood disorder, Risk stratification tool or intervention recommended, Patient-centric approach recommended
Garcia-Gonzalez et al., 2008	Cross-sectional survey (N = 102)	USA	Rheumatoid arthritis and systemic lupus erythematosus	Ethnicity, Side-effects, Perceived medication ineffectiveness, Patient-centric approach
Harrold et al., 2010	Interview (N = 26)	USA	Gout	Side-effects, Perceived medication ineffectiveness, Lack of motivation or social support, Cost, Risk stratification tool or intervention recommended, Patient-centric approach recommended
Harrold et al., 2009	Retrospective database (N = 4166)	USA	Gout	Age (older more adherent), Comorbid conditions, Risk stratification tool or intervention recommended, Patient-centric approach recommended
Hetland et al., 2012	Retrospective database, questionnaire (N = 2326)	Denmark	Rheumatoid arthritis	Age (younger more adherent), Side effects, Perceived medication ineffectiveness
Horne et al., 2009	Cross sectional survey (N = 1871)	USA	Inflammatory bowel disease	Age (older more adherent), Disease duration, Patient does not understand treatment, Side-effects, Forgetfulness or inconvenience, Dose regimen, Perceived mediation ineffectiveness Disease severity, Risk stratification tool or intervention recommended, Patient-centric approach recommended
Hughes et al., 2011	Feasibility survey (N = 112)	United Kingdom	Rheumatoid arthritis	Forgetfulness or inconvenience, Risk stratification tool or intervention recommended
Julian et al., 2009	Retrospective database, Telephone survey (N = 834)	USA	Systemic lupus erythematosus	Disease duration, Forgetfulness or inconvenience, Dose regimen, Mood disorder, Risk stratification tool or intervention recommended, Patient-centric approach recommended
Koneru et al., 2008	Retrospective database, questionnaire (N = 63)	USA	Systemic lupus erythematosus	Ethnicity, Patient does not understand treatment, Forgetfulness or inconvenience, Dose regimen, Forgetting instructions, Mood disorder, Lack of motivation or social support, Comorbid conditions, Cost, Risk stratification tool or intervention recommended, Patient-centric approach recommended
Kamperidis et al., 2012	Retrospective database (N = 238)	United Kingdom	Inflammatory bowel disease	Age (younger less adherent), Comorbid conditions, Risk stratification tool or intervention recommended
Lakotos et al., 2010	Questionnaire (N = 655)	Hungary	Inflammatory bowel disease	Side effects, Forgetfulness or inconvenience, Dose regimen, Risk stratification tool or intervention recommended, Patient-centric approach recommended
Li et al., 2010	Retrospective database	USA	Rheumatoid arthritis	Ethnicity, Side effects, Dose regimen
Lorish et al., 1990	Interview (N = 140)	United Kingdom	Rheumatoid arthritis	Patient does not understand treatment, Side effects, Forgetfulness or inconvenience, Dose regimen, Perceived mediation ineffectiveness, Risk stratification tool or intervention recommended, Patient-centric approach recommended
Marengo et al., 2012	Questionnaire, laboratory testing, electronic monitoring (N = 78)	USA	Systemic lupus erythematosus	Patient does not understand treatment, Dose regimen, Mood disorder, Comorbid conditions, Risk stratification tool or intervention, recommended, Patient-centric approach recommended
Muller et al., 2012	Questionnaire (N = 1199)	Estonia	Rheumatoid arthritis	Age (younger less adherent), Patient does not understand treatment, Side effects, Forgetting instructions, Patient-centric approach recommended
Moshkovska et al., 2009	Questionnaire, laboratory testing (N = 169)	United Kingdom	Ulcerative colitis	Age (younger less adherent), Ethnicity Side effects, Risk stratification tool or intervention recommended, Patient-centric approach recommended
Nahon et al., 2012	Questionnaire (N = 1663)	France	Inflammatory bowel disease	Mood disorder, Risk stratification tool or intervention recommended
Nguyen et al., 2009	Retrospective database, Cross sectional (N = 235)	USA	Inflammatory bowel disease	Age (younger less adherent), Ethnicity, Risk stratification tool or intervention recommended, Patient-centric approach recommended
Pascual-Ramos et al., 2009	Retrospective database, interview (N = 75)	Mexico	Rheumatoid arthritis	Age (older less adherent), Forgetting instructions, Patient-centric approach recommended
Richards et al., 2012	Retrospective database (N = 1372)	USA	Rheumatoid arthritis	Ethnicity, Dose regimen, Risk stratification tool or intervention recommended
Tuncay et al., 2007	Questionnaire (N = 100)	Turkey	Rheumatoid arthritis	Age (younger less adherent), Disease severity, Risk stratification tool or intervention recommended, Patient-centric approach recommended
Zwikker et al., 2012	A multidisciplinary tasks group	The Netherlands	Rheumatoid arthritis	Patient does not understand treatment, Side effects, Lack of motivation or social support, Risk stratification tool or intervention recommended, Patient-centric approach recommended

#### Analysis strategy (*Present*)

Following the reliability check, coding was synthesized to formulate a holistic set of findings and insights that are specific to understanding why, despite proven efficacy of treatment, autoimmunology patients are typically non-adherent.

## Results

The characteristics of studies varied and included retrospective database analyses, patient surveys, cross-sectional assessments, longitudinal studies, questionnaires, patient interviews, feasibility surveys, telephone surveys, laboratory tests, multidisciplinary task groups and electronic monitoring. Type of study also varied, with six quantitative, 12 qualitative and nine mixed-method studies (Tables 2 and 3). This variation contributed to the aim of this study as strong common themes emerged across vastly different study designs.

**Table 2:** Non-modifiable Risk Factors.

	Total Number of Studies	Age	Ethnicity	Disease Duration	Gender
Quantitative Studies (n) %	6	(2) 33%	(2) 33%	(2) 33%	(0) 0%
Qualitative Studies (n) %	12	(4) 33%	(3) 23%	(1) 8%	(1) 8%
Mixed Studies (n) %	9	(4) 44%	(4) 44%	(2) 22%	(1) 11%
**Total (n) %**	**27**	**(10) 37%**	**(9) 33%**	**(5) 19%**	**(2) 7%**

**Table 3:** Modifiable Risk Factors.

	Total Studies	Patient does not understand treatment	Side effects	Dose regimen	Perceived medication ineffectiveness	Forgetfulness or inconvenience	Disease severity	Comorbid condition	Presence of mood disorder	Lack of motivation or social support	Forgetting instructions	Cost
Quantitative Studies (n) %	6	(2) 33%	(1) 17%	(3) 50%	(1) 17%	(0) 0%	(1) 17%	(4) 67%	(1) 17%	(0) 0%	(0) 0%	(0) 0%
Qualitative Studies (n) %	12	(6) 50%	(8) 67%	(3) 25%	(4) 33%	(6) 50%	(3) 25%	(0) 0%	(2) 17%	(3) 25%	(1) 8%	(1) 8%
Mixed Method Studies (n) %	9	(4) 44%	(2) 22%	(3) 33%	(4) 44%	(2) 22%	(2) 22%	(2) 22%	(3) 33%	(1) 11%	(2) 22%	(1) 11%
**Total (n) %**	**27**	**(12) 44%**	**(11) 41%**	**(9) 33%**	**(9) 33%**	**(8) 30%**	**(6) 22%**	**(6) 22%**	**(6) 22%**	**(4) 15%**	**(3) 11%**	**(2) 7%**

Studies were published between 1990 and 2012. Twelve studies were based on study populations within North America (11 USA, one Mexico), 13 studies were based on Western/Eastern Europe and Middle East populations (10 Western Europe, two Eastern Europe and one Middle East), and two studies were based on AustralAsia populations (two New Zealand). Twelve studies focused on RA patients, six in IBD, five in SLE, three in Gout, and one in UC.

**Fig 2:**
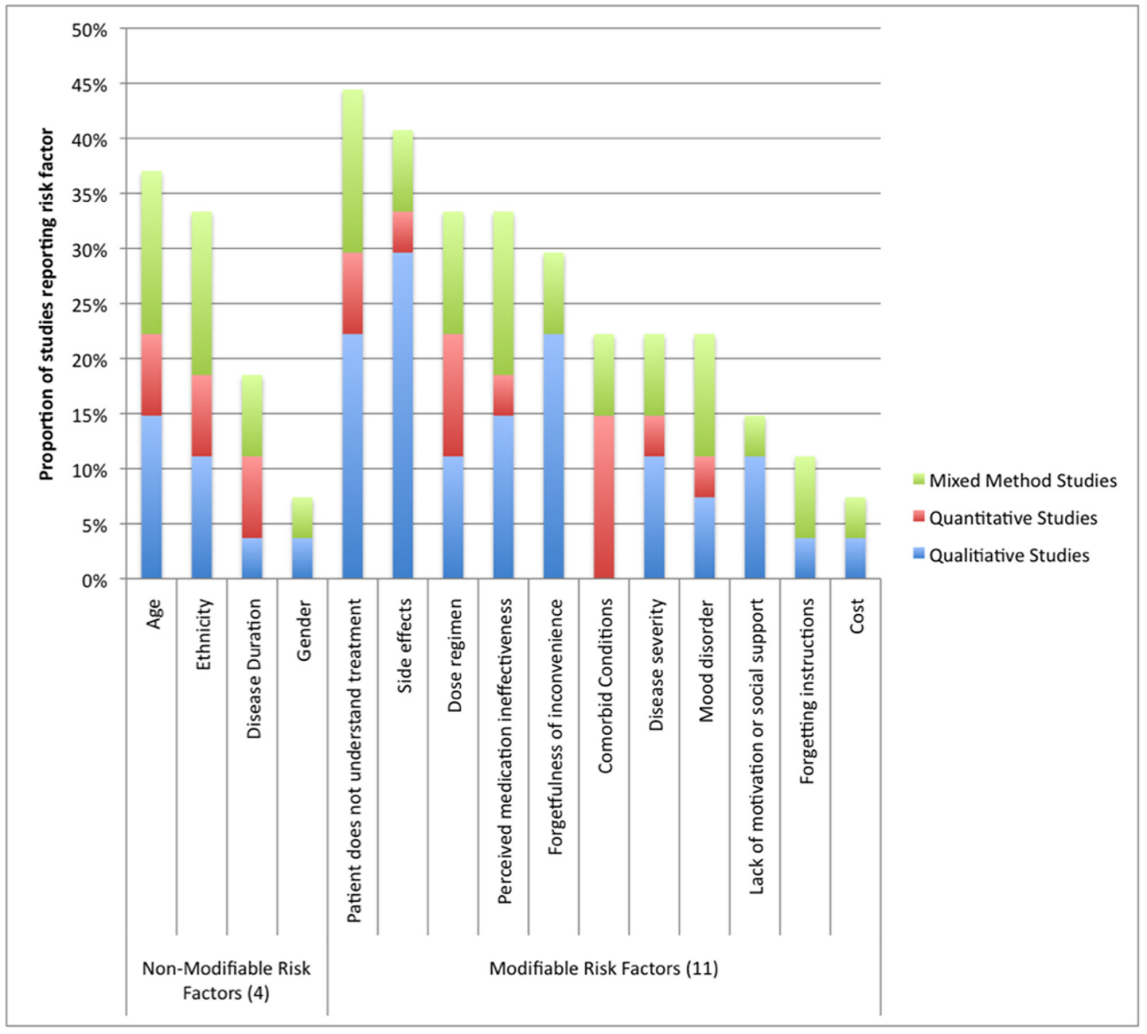
Proportion of studies reporting modifiable and non-modifiable risk factors.

Out of the 27 studies, four non-modifiable and 11 modifiable risk factors were uncovered (Fig 2. and Tables 2 and 3). The four non-modifiable risk factors were age, race/ethnicity, gender, and disease duration. The 11 modifiable risk factors included patients not understanding treatment, side effects / adverse events, forgetfulness / inconvenience, dose regimen, forgetting instructions, medication ineffectiveness, presence of a mood disorder, lack of motivation or social support, disease severity, cost, and presence of a comorbid condition.

Across all study types and indications, the most commonly described determinants of medication non-adherence, confirmed in ≥33% of studies, were patients not understanding treatment (44%), side effects (41%), age (37%), dose regimen (33%), forgetfulness/inconvenience (33%), perceived medication ineffectiveness (33%) and ethnicity (33%).

Specifically looking at the 37% of studies reporting age as a non-modifiable factor, 30% indicated that adherence increased as age progressed, while 7% indicated that younger people were more adherent (Table 4).

**Table 4:** Sub-set of age.

	Total Number of Studies (N)	Age	Younger age more adherent	Older age more adherent
Number of Quantitative Studies (n) %	6	(2) 33%	(0) 0%	2 (33%)
Number of Qualitative Studies (n) %	12	(4) 33%	(0) 0%	4 (31%)
Number of Mixed Studies (n) %	9	(4) 44%	2 (22%)	2 (22%)
**Total (n) %**	**27**	**(10) 37%**	**2 (7%)**	**8 (30%)**

Limiting studies to US-based populations only (n = 11), the most common modifiable factors (reported in ≥33% of studies) were dose regimen (55%), and patients not understanding treatment (45%), with side effects and perceived medication ineffectiveness at 36%. Ethnicity (55%) was the most common non-modifiable factor.

The most common factors (reported in ≥50% of studies) in RA specific studies conducted in the USA (n = 4) were dose regimen (75%), ethnicity (50%), patients not understanding treatment (50%) and co-morbid conditions (50%).

An unexpected finding was that the vast majority of study authors overwhelmingly advocated for the development of risk stratification tools (81% of reported studies) with a patient-centric approach (67% of reported studies) (Table 5).

**Table 5:** Recommended Treatment Approaches.

	Total Number of Studies (N)	Articles advocating for the need for a risk stratification tool or intervention	Articles advocating for a patient-centric approach to treatment for non-adherence
Quantitative Studies (n) %	6	(5) 83%	(2) 33%
Qualitative Studies (n) %	12	(10) 83%	(10) 83%
Mixed Studies (n) %	9	(7) 78%	(6) 67%
**Total (n) %**	**27**	**(22) 81%**	**(18) 67%**

Despite variations in study characteristics, year, location and differing target groups, recommendations for risk-stratification tools and a patient-centric approach appeared in the majority of studies.

## Discussion

As we uncover the factors leading to non-adherence, we can begin to build profiles and digitally tailor content. Past research has shown that clinical characteristics can be used to maximize website utilization, and that short, tailored exercises may attract a wider audience [62]. However, this has not been attempted in autoimmunology.

Three of the five modifiable risk factors (not understanding treatment, perceived medication ineffectiveness and forgetfulness/inconvenience) could be addressed through targeted patient communications, shared decision making tools, or more regular patient interaction [63]. The relationship between side effects and non-adherence is longstanding across many therapeutic areas [64–70], and might be a central focus of program content (e.g. support group discussions or text messages).

Forgetfulness and inconvenience were frequently cited in the literature (33% of studies). While text messaging and email reminders could manage general forgetfulness, auto-immune diseases are serious and often painful, so it is most likely important to consider behavioral motivations behind non-adherence. Immunological disorders and medicine are understandably inconvenient, however as noted by the WHO [23], the long-term consequences of non-adherence compromise patient safety and quality of life. Digital tools should be not be paternalistic, and allow patients to make cost-benefit decisions in collaboration with their physicians.

Five studies found that non-adherence increased as the frequency of dosing increased [44,71–74], two studies indicated that non-adherence increased as regimens became more complex [47,75], and one found a negative relationship between adherence and duration of therapy [76]. In 2010, Li et al found that infusion patients were more adherent than those on injection schedules, and also attributed increased adherence to decreased frequency of administration and clinical assistance from physicians or nurses [45]. From these results it is clear that digital interventions need to be tailored to medication dose frequency.

In the past, significant effort to address non-adherence has been placed on reducing costs to patients and providing patients with detailed instructions on drug application. However, our findings indicate that cost (7%) and forgetting instructions (11%) were less impactful than other modifiable factors. The impact of cost on an individual or families’ decision to pursue treatment is difficult to measure, especially in the complex US market where there is a paucity of empirical evidence explaining how demand and supply prices influence utilization [77]. Interventions that focus on how and when to administer medication have also proven to be ineffective [78]. Based on our results, the impact of efforts spent at circumventing cost (downloadable coupons, assistance programs) and providing instructions (printouts, detail aids) should be reassessed, or be considered as part of a holistic framework of activities designed to address the issue of non-adherence.

Given the tremendous health and economic costs attributed to medication non-adherence in autoimmune diseases, the vast majority of publications (81%) identified the need for risk stratification tools or interventions that are patient–centric (67%). Risk stratification tools exist today, but they are largely physician-focused and have yet to be digitized. Some examples typically used in autoimmunology are the Compliance Questionnaire Rheumatology [79], the Medication Adherence Rating Scale [80], the Morisky Medication Adherence Scale [81] and the Rheumatoid Arthritis Severity Scale [82]. As in other condition areas, these standardized tools and assessments can be modified to provide targeted feedback for both patients and providers [59,78,83–85].

However, careful consideration of process and design must be considered when developing patient-centric interventions. Past attempts in autoimmunology showed that physician awareness alone was necessary but not sufficient to improve adherence [86], and interventions may have no impact on patient adherence or quality of life [58].

### Limitations and Future Considerations

Limitations are similar to other research employing a GT approach. Only non-adherence risk factors reported in the literature were included and categorized; risk factors not listed within the works may very well exist and outweigh the factors outlined in this study. Regardless, these study results summarize the risk factors supported by the current evidence base and can be easily articulated to experts in non-medical occupations such as website designers, project managers marketers.

Another possible limitation is the inclusion of both quantitative and qualitative studies; modifiable risk factors such as forgetfulness or lack of motivation or social support cannot be measured quantitatively. However, despite this limitation it is important to note that forgetfulness or inconvenience was a top modifiable risk factor, and its overall impact is most likely underrepresented.

A significant strength of this study can be found in the GT approach. The study began with no pre-conceived biases, and findings are reflective of determinants of non-adherence deemed important by experts in the field and are evidence based. The relative consistency of top non-modifiable and modifiable risk factors is noteworthy. This study is replicable, and future studies could be undertaken to measure consistency of findings.

Finally, the intention of this research was not to provide conclusive evidence that identifies all determinants of non-adherence for autoimmune diseases. Rather, it is a first step in recognizing common risk factors that can be applied to digital tool development.

## Conclusions

According to these findings, adherence outcomes and digital attrition in autoimmunology patients could be improved if patients were given tailored tools to help them gain greater understanding of their disease, the role of medications, and information on side effects, and the role of dose regimen.

Results also indicate that rather than adopting a traditional systems level approach, digital health might focus on cohorts with heterogeneous needs, and develop tailored interventions based on individual non-adherence patterns.

Digital health programs focusing on daily diaries, stage-based modules, standardized reminders and emails have only proven to be moderately successful. A patient’s relationship to treatment is highly personal, and digital interventions should take advantage of technologies that enable tailoring for the unique needs of individuals. Digital health programs addressing other conditions have shown promise, and our evidence indicates that autoimmune patient cohorts may benefit from tailored and accessible electronic resources.

## Supporting Information

S1 PRISMA Checklist PRISMA Checklist. (DOC)
